# Fauna Europaea: Mollusca – Bivalvia

**DOI:** 10.3897/BDJ.3.e5211

**Published:** 2015-07-17

**Authors:** Rafael Araujo, Yde de Jong

**Affiliations:** ‡ Museo Nacional de Ciencias Naturales, Madrid, Spain; § University of Amsterdam - Faculty of Science, Amsterdam, Netherlands; | Museum für Naturkunde, Berlin, Germany

**Keywords:** Biodiversity Informatics, Fauna Europaea, Taxonomic indexing, Zoology, Biodiversity, Taxonomy, Mollusca, Bivalvia, Margaritiferidae, Unionidae, Sphaeriidae, Cyrenidae, Dreissenidae, freshwater mussels, zebra mussel

## Abstract

*Fauna Europaea* provides a public web-service with an index of scientific names (including important synonyms) of all living European land and freshwater animals, their geographical distribution at country level (up to the Urals, excluding the Caucasus region), and some additional information. The *Fauna Europaea* project covers about 230,000 taxonomic names, including 130,000 accepted species and 14,000 accepted subspecies, which is much more than the originally projected number of 100,000 species. This represents a huge effort by more than 400 contributing specialists throughout Europe and is a unique (standard) reference suitable for many users in science, government, industry, nature conservation and education. For the Mollusca-Bivalvia, data from 5 families (Margaritiferidae, Unionidae, Sphaeriidae, Cyrenidae, Dreissenidae) containing 55 species are included in this paper.

European freshwater bivalves belong to the Orders Unionoida and Cardiida. All the European unionoids are included in the superfamily Unionoidea, the freshwater mussels or naiads. The European cardiids belong to the following three superfamilies: Cardioidea, Cyrenoidea and Dreissenoidea. Among the Unionoidea there are the most imperilled animal groups on the planet while the Cardioidea includes the cosmopolitan genus *Pisidium*, the Cyrenoidea the Asiatic clam (*Corbicula fluminea*) and the Dreissenoidea the famous invasive zebra mussel (*Dreissena polymorpha*). Basic information is summarized on their taxonomy and biology. Tabulations include a complete list of the current estimated families, genera and species.

## Introduction

The European Commission published the European Community Biodiversity Strategy, providing a framework for development of Community policies and instruments in order to comply with the Convention on Biological Diversity. This Strategy recognises the current incomplete state of knowledge at all levels concerning biodiversity, which is a constraint on the successful implementation of the Convention. Fauna Europaea contributes to this Strategy by supporting one of the main themes: to identify and catalogue the components of European biodiversity into a database in order to serve as a basic tool for science and conservation policies.

With regard to biodiversity in Europe, both science and policies depend on a knowledge of its components. The assessment of biodiversity, monitoring changes, sustainable exploitation of biodiversity, and much legislative work depend upon a validated overview of taxonomic biodiversity. Towards this end Fauna Europaea plays a major role, providing a web-based information infrastructure with an index of scientific names (including important synonyms) of all living European land and freshwater animals, their geographical distribution at country level and some additional useful information. In this sense, the Fauna Europaea database provides a unique reference for many user-groups such as scientists, governments, industries, conservation communities and educational programs.

Fauna Europaea started in 2000 as an EC-FP5 four-years project, delivering its first release in 2004 (Jong et al. 2014). After thirteen years of steady progress, in order to efficiently disseminate the Fauna Europaea results and to increase the acknowledgement of the Fauna Europaea contributors, novel e-Publishing tools have been applied to prepare data-papers of all major taxonomic groups. For this purpose a special Biodiversity Data Journal Series has been compiled, called Contributions on Fauna Europaea. This work was initiated during the ViBRANT project and is further supported by the recently started EU BON project. This paper holds the first publication of the Fauna Europaea Mollusca-Bivalvia data sector as a BDJ data paper.

Within the EU BON project also further steps will be made to implement *Fauna Europaea* as a basic tool and standard reference for biodiversity research and to evaluate taxonomic expertise capacity in Europe. The *Fauna Europaea* data-papers will contribute to a quality assessement on biodiversity data by providing estimates on gaps in taxonomic information and knowledge.

## General description

### Purpose

The Fauna Europaea is a database of the scientific names and distribution of all living, currently known multicellular European land and fresh-water animal species assembled by a large network of experts, using advanced electronic tools for data collations and validation routines. An extended description of the Fauna Europaea project can be found in Jong et al. 2014. A summary is given in the sections below.

**Figure 1.**
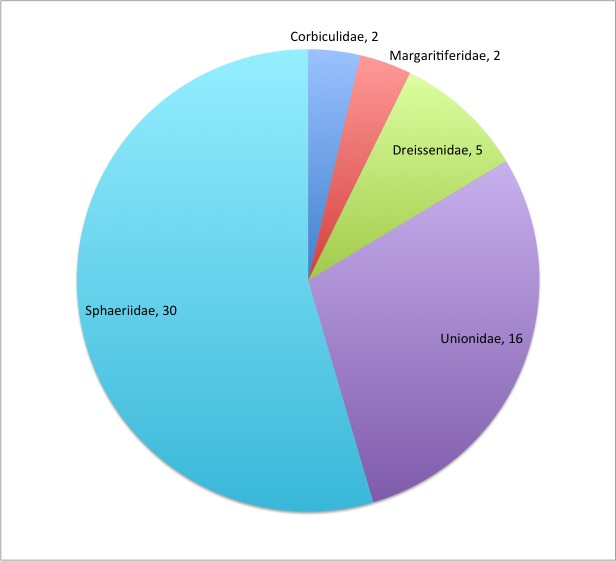
aEu Mollusca-Bivalvia species per family. See Table 1 for family statistics.

The Mollusca-Bivalvia are one of the 58 Fauna Europaea major taxonomic groups, covering 55 species (Fig. 1).

### Additional information

**(Introduction Mollusca-Bivalvia**

Bivalves (8,000 species) are the second richest mollusc class after the Gastropoda (60,000 species). Freshwater bivalves live in rivers and lakes around the world except Antarctica. They represent three subclasses, 19 families, 206 genera and about one thousand species (Bogan 2008). European freshwater bivalves belong to the orders Unionoida and Cardiida (Carter et al. 2011). All the European unionoids are included in the superfamily Unionoidea, the freshwater mussels or naiads. The European cardiids belong to the following three superfamilies: Cardioidea, Cyrenoidea and Dreissenoidea. Among the Unionoidea (families Margaritiferidae and Unionidae) there are the most imperilled animal groups on the planet while the Cardioidea includes the cosmopolitan genus *Pisidium* (family Sphaeriidae); the Cyrenoidea includes the Asiatic clam (*Corbicula fluminea*) (family Cyrenidae) and the Dreissenoidea includes the famous invasive zebra mussel (*Dreissena polymorpha*) (family Dreissenidae). Most freshwater bivalves are filter or/and suspension feeders, living buried in the substratum where they can move thanks to their muscular foot and can clear large quantities of water. Sphaeriids can creep among weeds and rocks and unionoids can move few meters in one day. The zebra mussels live adhered to the substratum by a byssus.

**Family Sphaeriidae**

**Figure 2.**
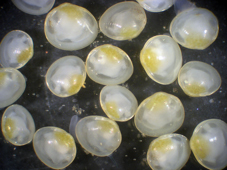
Living specimens of *Pisidium nitidum*.

Bogan 2008 recognizes 34 species of this family in the Palearctic. Species identification in European Sphaeriidae (Fig. 2) can be as difficult as in Unionoida, and the miniaturization of the species of the Family Sphaeriidae can complicate the taxonomic task. In this group, the shell shape, position of the umbos, sculpture, tumidity, porosity, texture, lustre and thickness should be useful characters, although the hinge gives normally the principal key for a correct identification. The most important diagnostic hinge characters are: shape of cardinal teeth, shape and length of lateral teeth, length of the hinge plate (relative to overall shell length), shape of the ligament pit and the presence or absence of callus (Ellis 1978, Killeen et al. 2004). Molecular taxonomy has not yet reached the European sphaeriids as has happened with other faunas (Lee and Foighil 2003).

Pea clams (Sphaeriidae) are ubiquitous in freshwater ecosystems; they have a minimal size of 2 mm and a life span of one year. They are always hermaphrodite and incubate the fertilized eggs into their gills until the juvenile stage (Araujo and Ramos 1997, Araujo and Ramos 1999).

**Family Cyrenidae**

**Figure 3.**
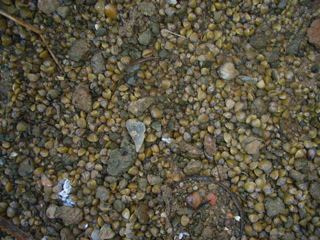
*Corbicula fluminea* covering the river bottom.

Often also named by its synomy Corbiculidae. In Europe only lives the genus *Corbicula* (). The number of species considered under this genus is not yet known, we recognize two hyper variable species, *Corbicula fluminea* (Müller, 1774) (Fig. 3) and *Corbicula fluminalis* (Müller, 1774), although their taxonomical status is not clear. In the 20th and 21th centuries *Corbicula* clams were introduced in North America, South America, Europe and North Africa. Their populations are also hermaphrodite with two different reproductive strategies: incubate the fertilized eggs into their gills until the juvenile stage or can present planktonic larvae (Korniushin and Glaubrecht 2002).

**Family Dreissenidae**

**Figure 4.**
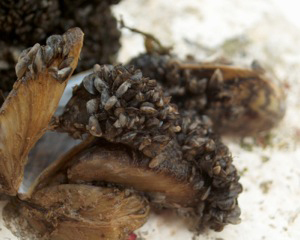
Colony of zebra mussel, *Dreissena polymorpha*.

In Europe live two genera and two species, *Dreissena polymprpha* (Fig. 4) and *Mytilopsis leucophaeta*, both famous invasive bivalves. Although they are considered freshwater molluscs, they can survive in brackish water, for example *Dreissena* in the Black and Caspian seas or *Mytilopsis leucophaeta* in North America, where autochthonous populations live in ecological equilibrium (Heiler et al. 2010, Kennedy 2011). Since the XIX century, the zebra mussel has spread to the European continent helped by river transport, as the first mass invasion of a Ponto-Caspian species in Europe (Bij de Vaate et al. 2002). In 1980s, mussels transported on cargo ships caused the spread of *D. polymorpha* to the North American continent. Species on this family can be identified by its mytiliform form, sometimes quadrate, with the hinge edentulous and the umbos anterior or terminal. The umbo cavity is bridged by a septum or myophore. They have a byssus which allows them to form dense colonies. They have separated sexes and planktonic larvae.

Recentrly Bilandzija (Bilandžija et al. 2013) have described two new species of the genus *Congeria*. In this way, the family Dreissenidae in Europe also includes the genus *Congeria* with three species, *C. kusceri*, *C. jalcizi* and *C. mulaomerovici*, all living in caves of the Dinaric karst.

**Families Unionidae and Margaritiferidae**

**Figure 5.**
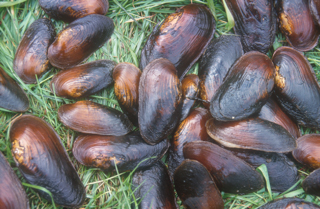
The endangered species *Margaritifera margaritifera*.

**Figure 6.**
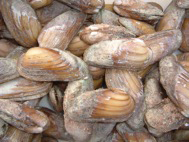
*Unio tumidiformis* lives only in some rivers of the South of Spain.

The assemblage fauna of European Unionoida includes only two familes, Margaritiferidae (Fig. 5) and Unionidae (Fig. 6), is not very diverse in comparison with other areas of the world, for instance the Nearctic, with 300 species and where a single river may have more species than all those in Europe. In the Palearctic there are recognized between 60 and 100 species of Unionoida (Graf 2010, Graf and Cummings 2013, Bogan 2008). Up to the beginning of the twentieth century, the number of described taxa in Europe was about 1,500 species due to an overestimation of species richness based on shell characters (Graf 2010). Since then, taxonomy and systematics of European freshwater mussels were reconstructed starting with the seminal paper from Haas (Haas 1969) who considered 58 taxa in the West Palearctic. Currently, this figure is changing reflecting the use of molecular taxonomic tools which are unrevealing previously hidden lineages (Araujo et al. 2009, Reis and Araujo 2009, Prié and Puillandre 2013). Here we consider 16 native species, belonging to two families and six genera, but the work in not yet finished, with many of the Haas (Haas 1969) subspecies needed to be clarified.

Freshwater mussels, also known as naiads, can grow to lengths of 25 cm and live more than a century. They are one of the most imperilled animal groups on the planet, yet they play an extremely important role in the ecology of freshwater ecosystems as a main component of the freshwater biomass (Vaughn and Hakenkamp 2001, Strayer et al. 2004). The dramatic changes taking place in freshwater ecosystems during the last century have played a part in the large-scale disappearance of these and other animals (Lydeard et al. 2004). Some species fulfil criteria of indicator, flagship and umbrella species, making them ideal targets in aquatic conservation, as it is the case of the freshwater pearl mussel in Europe *Margaritifera margaritifera* (Geist 2010).

In addition, one of the most amazing traits about freshwater mussels is their specialized reproductive strategy: the eggs are fertilized in the mussel gills (marsupium), where also occurs the segmentation until the glochidium, which has a temporary but obligatory parasitic stage in which the larvae (glochidia) attach to the external surface of a suitable host prior to metamorphosis to the free-living juvenile stage. The males release to the water the sperm, which will be siphoned for the females for fertilization (Kat 1984, Bauer 1987).

The form of the Unionoida shell can vary according to the biotype, the environmental influences giving rise to changes through which the identification of a shell can be made more difficult.

## Project description

### Title

This Biodiversity Data Journal (BDJ) data paper includes the taxonomic indexing efforts in the Fauna Europaea on European Mollusca-Bivalvia covering the first two versions of Fauna Europaea worked on between 2000 and 2013 (up to version 2.6).<br/>

### Personnel

The taxonomic framework of Fauna Europaea includes partner institutes, providing taxonomic expertise and information, and expert networks taking care about data collation.

Every taxonomic group is covered by at least one Group Coordinator responsible for the supervision and integrated input of taxonomic and distributional data of a particular group. The Fauna Europaea checklist would not have reached its current level of completion without the input from several groups of specialists. The formal responsibility of collating and delivering the data of relevant families rested with a number of Taxonomic Specialists (see Table 1). For Mollusca-Bivalvia the responsible Group Coordinator and Taxonomic specialist is Rafael Araujo. A more detail overview of the Fauna Europaea classification and expertise network for Mollusca-Bivalvia can be found here: http://www.faunaeur.org/experts.php?id=319.

Data management tasks are carried out by the *Fauna Europaea* project bureau. During the project phase (until 2004) a network of principal partners took responsability for various management tasks: Zoological Museum Amsterdam (general management & system development), Zoological Museum of Copenhagen (data collation), National Museum of Natural History in Paris (data validation) and Museum and Institute of Zoology in Warsaw (NAS extension). Once the formal end of the project ended (2004-2013) all tasks were were taken over by the Zoological Museum Amsterdam.

### Study area description

The area study covers the European mainland (Western Palearctic), including the Macaronesian islands, excluding the Caucasus, Turkey, Arabian Peninsula and Northern Africa (see: Geographic coverage).

### Design description

*Standards*. Group coordinators and taxonomic specialists have to deliver the (sub)species names according to strict standards. The names provided by Fauna Europaea are scientific names. The taxonomic scope includes issues like, (1) the definition of criteria used to identify the accepted species-group taxa, (2) the hierarchy (classification scheme) for the accommodation of the all accepted species and (3), relevant synonyms, and (4) the correct nomenclature. The Fauna Europaea 'Guidelines for Group Coordinators and Taxonomic Specialists', include the standards, protocols, scope, and limits that provide the instructions for all more then 400 specialists contributing to the project, strictly following the provisions of the current edition of the International Code of Zoological Nomenclature.

*Data management*. The data records could either be entered offline into a preformatted MS-Excel worksheet or directly into the Fauna Europaea transaction database using an online browser interface. Since 2013 the data servers are hosted at the Museum für Naturkunde in Berlin (migrated from ZMA-UvA).

*Data set*. The Fauna Europaea basic data set consists of: accepted (sub)species names (including authorship), synonym names (including authorship), a taxonomic hierarchy/classification, misapplied names (including misspellings and alternative taxonomic views), homonym annotations, expert details, European distribution (at country level), Global distribution (only for European species), taxonomic reference (optional), occurrence reference (optional).

### Funding

*Fauna Europaea* was funded by the European Commission under the Fifth Framework Programme and contributed to the Support for Research Infrastructures work programme with Thematic Priority Biodiversity (EVR1-1999-20001) for a period of four years (1 March 2000 - 1 March 2004), including a short 'NAS extension', allowing EU candidate accession countries to participate. Follow-up support was given by the EC-FP5 EuroCAT project (EVR1-CT-2002-20011), by the EC-FP6 ENBI project (EVK2-CT-2002-20020), by the EC-FP6 EDIT project (GCE 018340), by the EC-FP7 PESI project (RI-223806) and by the EC-FP7 ViBRANT project (RI-261532). Continuing management and hosting of the Fauna Europaea services was supported by the University of Amsterdam (Zoological Museum Amsterdam) and SARA/Vancis. Recently the hosting of Fauna Europaea was taken over by the Museum für Naturkunde in Berlin, supported by the EC-FP7 EU BON project (grant agreement №308454).<br/>

For preparing the Mollusca-Bivalvia data set additional support was received from the Fauna Ibérica Project (Museo Nacional de Ciencias Naturales-C.S.I.C).

## Sampling methods

### Study extent

See spatial coverage and geographic coverage descriptions.

### Sampling description

Fauna Europaea data have been assembled by principal taxonomic experts, based on their individual expertise, including literature sources, collection research, and field observations. In total no less than 476 experts contributed taxonomic and/or faunistic information for Fauna Europaea. The vast majority of the experts are from Europe (including EU non-member states). As a unique feature, Fauna Europaea funds were set aside for rewarding/compensating for the work of taxonomic specialists and group coordinators.

**Figure 7.**
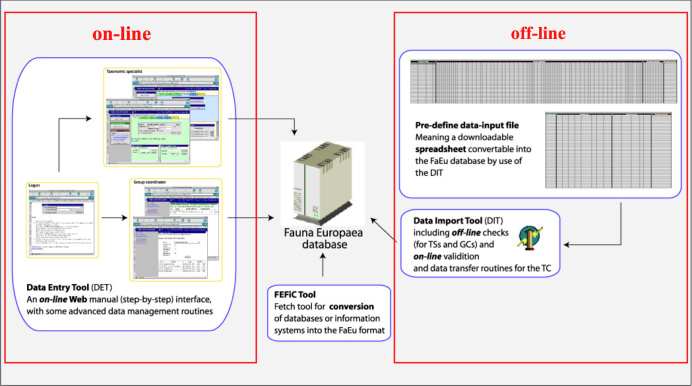
Fauna Europaea on-line (browser interfaces) and off-line (spreadsheets) data entry tools.

To facilitate data transfer and data import, sophisticated on-line (web interfaces) and off-line (spreadsheets) data-entry routines were built, integrated within an underlying central Fauna Europaea transaction database (see Fig. 7). This includes advanced batch data import routines and utilities to display and monitor the data processing within the system. In retrospect, it seems that the off-line submission of data was probably the best for bulk import during the project phase, while the on-line tool was preferred to enter modifications in later versions. This system works well but will be replaced in 2013.

A first release of the Fauna Europaea index via the web-portal has been presented at 27^th^ of September 2004, the most recent release (version 2.6.2) was launched at 29 August 2013. An overview of Fauna Europaea releases can be found here: http://www.faunaeur.org/about_fauna_versions.php.

### Quality control

Fauna Europaea data are unique in a sense that they are fully expert based. Selecting leading experts for all groups assured the systematic reliability and consistency of the Fauna Europaea data.

Furthermore, all Fauna Europaea data sets are intensively reviewed at regional and thematic validation meetings, at review sessions on taxonomic symposia (for some groups), by Fauna Europaea Focal Points (during the FaEu-NAS and PESI projects) and by various end-users sending annotations using the web form at the web-portal. Additional validation on gaps and correct spelling was effected at the validation office in Paris.

Checks on technical and logical correctness of the data have been implemented in the data entry tools, including around 50 "Taxonomic Integrity Rules". This validation tool proved to be of huge value for both the experts and project management, and contributed significantly to preparation of a remarkably clean and consistent data set. This thorough reviewing makes Fauna Europaea the most scrutinised data sets in its domain.

Estimated gaps for Mollusca-Bivalvia, in terms of described species that are known from Europe, but currently not included in the database are presented in Table 1. They range from zero for most families up to about 5%. The information represented in this group will be updated on short term, mainly regarding the already accepted Unionidae species *Unio ravoisieri* Deshayes, 1847 living only in North East Spain and included at the National Catalogue as Endandered. Indeed, other new Unionidae taxa will be added once the new collected material from Italy, Croatia, Albania and Greece is studied. Most probably some of the subspecies here cited in these areas will be considered species in a near future (Araujo *et al*. in prep.). This expected endemism at the European East peninsulas should be similar to the one recently found in the Iberian Peninsula, where the geographically restricted species *Unio delphinus* Spengler, 1793 and *U. tumidiformis* Castro, 1885 have been recently redescribed (Araujo et al. 2009, Reis and Araujo 2009).

To optimise the use and implementation of a uniform and correct nomenclature, also following the global efforts on establishing a so-called 'Global Names Architecture' (Pyle and Michel 2008, Patterson et al. 2010), a cross-referencing of the Fauna Europaea Mollusca – Bivalvia data-set with relevant nomenclators, including the Mollusks content of AnimalBase, is recommended as well as a connection with relevant name services and checklists, like CLECOM (see also Additional information).

### Step description

By evaluating team structure and life cycle procedures (data-entry, validation, updating, etc.), clear definitions of roles of users and user-groups, according to the taxonomic framework were established, including ownership and read and writes privileges, and their changes during the project life-cycle. In addition, guidelines on common data exchange formats and codes have been issued (see also the 'Guidelines for Experts' document).

## Geographic coverage

### Description

**Figure 8.**
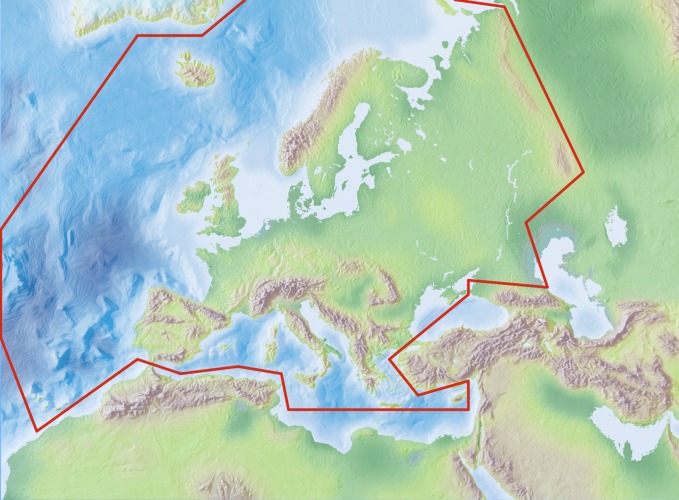
Fauna Europaea geographic coverage ('minimal Europe').

Species and subspecies distributions in Fauna Europaea are registered at least a country level, i.e. for political countries. For this purpose the FaEu geographical system basically follows the TDWG standards. The covered area includes the European mainland (Western Palearctic), plus the Macaronesian islands (excl. Cape Verde Islands), Cyprus, Franz Josef Land and Novaya Zemlya. Western Kazakhstan and the Caucasus are excluded (see Fig. 8).

The focus is on species (or subspecies) of European multicellular animals of terrestrial and freshwater environments. Species in brackish waters, occupying the marine/freshwater or marine/terrestrial transition zones, are generally excluded. Nevertheless, we have considered some bivalves which can survive in brackish waters, as Mytilopsis and Dreissena.

### Coordinates

Mediterranean (N 35°) and Arctic Islands (N 82°) Latitude; Atlantic Ocean (Mid-Atlantic Ridge) (W 30°) and Ural (E 60°) Longitude.

## Taxonomic coverage

### Description

The Fauna Europaea database contains the scientific names of all living European land and freshwater animal species, including numerous infra-groups and synonyms. More details about the conceptual background of Fauna Europaea and standards followed are described above and in the project description paper(s).

This data paper covers the Mollusca-Bivalvia content of Fauna Europaea, including 5 families, 55 species, 34 subspecies and 96 (sub)species synonyms (see Fig. 1).

Although the classification used in FaunaEuropaea include the order Veneroida with the superfamilies Cardioidea, Corbiculoidea, Sphaerioidea and Dreissenoidea, now it has changed to the order Cardiida with 3 superfamilies: Cardioidea, Cyrenoidea and Dreissenoidea. Indeed, family Corbiculidae is now Cyrenidae (Carter et al. 2011).

### Taxa included

**Table:** 

Rank	Scientific Name	Common Name
kingdom	Animalia	
subkingdom	Eumetazoa	
phylum	Mollusca	
class	Bivalvia	
subclass	Eulamellibranchia	
superorder	Heterodonta	
order	Veneroida	
superfamily	Cardioidea	
superfamily	Corbiculoidea	
family	Corbiculidae	
superfamily	Dreissenoidea	
family	Dreissenidae	
superfamily	Sphaerioidea	
family	Sphaeriidae	
superorder	Palaeoheterodonta	
order	Unionoida	
superfamily	Unionoidea	
family	Margaritiferidae	
family	Unionidae	
subfamily	Unioninae	

## Temporal coverage

**Living time period:** Currently living.

### Notes

Currently living animals in stable populations, largely excluding (1) rare/irregular immigrants, intruder or invader species, (2) accidental or deliberate releases of exotic (pet) species, (3) domesticated animals, (4) foreign species imported and released for bio-control or (5) foreign species largely confined to hothouses.

## Usage rights

### Use license

Open Data Commons Attribution License

### IP rights notes

*Fauna Europaea* data are licensed under CC BY SA version 4.0. The property rights of experts over their data is covered by their Fauna Europaea contract agreements. For more IPR details see: http://www.faunaeur.org/copyright.php.

## Data resources

### Data package title

Fauna Europaea - Mollusca-Bivalvia

### Resource link

http://www.faunaeur.org/Data_papers/FaEu_Mollusca-Bivalvia_2.6.2.zip

### Alternative identifiers

http://www.faunaeur.org/full_results.php?id=11480

### Number of data sets

2

### Data set 1.

#### Data set name

Fauna Europaea - Mollusca-Bivalvia version 2.6.2 - species

#### Data format

CSV

#### Number of columns

25

#### Character set

UTF-8

#### Download URL

http://www.faunaeur.org/Data_papers/FaEu_Mollusca-Bivalvia_2.6.2.zip

#### Description

**Data set 1.** 

Column label	Column description
datasetName	The name identifying the data set from which the record was derived (http://rs.tdwg.org/dwc/terms/datasetName).
version	Release version of data set.
versionIssued	Issue data of data set version.
rights	Information about rights held in and over the resource (http://purl.org/dc/terms/rights).
rightsHolder	A person or organization owning or managing rights over the resource (http://purl.org/dc/terms/rightsHolder).
accessRights	Information about who can access the resource or an indication of its security status (http://purl.org/dc/terms/accessRights).
taxonID	An identifier for the set of taxon information (http://rs.tdwg.org/dwc/terms/taxonID).
parentNameUsageID	An identifier for the name usage of the direct parent taxon (in a classification) of the most specific element of the scientificName (http://rs.tdwg.org/dwc/terms/parentNameUsageID).
scientificName	The full scientific name, with authorship and date information if known (http://rs.tdwg.org/dwc/terms/scientificName).
acceptedNameUsage	The full name, with authorship and date information if known, of the currently valid (zoological) taxon (http://rs.tdwg.org/dwc/terms/acceptedNameUsage).
originalNameUsage	The original combination (genus and species group names), as firstly established under the rules of the associated nomenclaturalCode (http://rs.tdwg.org/dwc/terms/originalNameUsage).
family	The full scientific name of the family in which the taxon is classified (http://rs.tdwg.org/dwc/terms/family).
familyNameId	An identifier for the family name.
genus	The full scientific name of the genus in which the taxon is classified (http://rs.tdwg.org/dwc/terms/genus).
subgenus	The full scientific name of the subgenus in which the taxon is classified. Values include the genus to avoid homonym confusion (http://rs.tdwg.org/dwc/terms/subgenus).
specificEpithet	The name of the first or species epithet of the scientificName (http://rs.tdwg.org/dwc/terms/specificEpithet).
infraspecificEpithet	The name of the lowest or terminal infraspecific epithet of the scientificName, excluding any rank designation (http://rs.tdwg.org/dwc/terms/infraspecificEpithet).
taxonRank	The taxonomic rank of the most specific name in the scientificName (http://rs.tdwg.org/dwc/terms/infraspecificEpithet).
scientificNameAuthorship	The authorship information for the scientificName formatted according to the conventions of the applicable nomenclaturalCode (http://rs.tdwg.org/dwc/terms/scientificNameAuthorship).
authorName	Author name information
namePublishedInYear	The four-digit year in which the scientificName was published (http://rs.tdwg.org/dwc/terms/namePublishedInYear).
Brackets	Annotation if authorship should be put between parentheses.
nomenclaturalCode	The nomenclatural code under which the scientificName is constructed (http://rs.tdwg.org/dwc/terms/nomenclaturalCode).
taxonomicStatus	The status of the use of the scientificName as a label for a taxon (http://rs.tdwg.org/dwc/terms/taxonomicStatus).
resourceDescription	An account of the resource, including a data-paper DOI (http://purl.org/dc/terms/description)

### Data set 2.

#### Data set name

Fauna Europaea - Mollusca-Bivalvia version 2.6.2 - hierarchy

#### Data format

CSV

#### Number of columns

12

#### Character set

UTF-8

#### Download URL

http://www.faunaeur.org/Data_papers/FaEu_Mollusca-Bivalvia_2.6.2.zip

#### Description

**Data set 2.** 

Column label	Column description
datasetName	The name identifying the data set from which the record was derived (http://rs.tdwg.org/dwc/terms/datasetName).
version	Release version of data set.
versionIssued	Issue data of data set version.
rights	Information about rights held in and over the resource (http://purl.org/dc/terms/rights).
rightsHolder	A person or organization owning or managing rights over the resource (http://purl.org/dc/terms/rightsHolder).
accessRights	Information about who can access the resource or an indication of its security status (http://purl.org/dc/terms/accessRights).
taxonName	The full scientific name of the higher-level taxon
scientificNameAuthorship	The authorship information for the scientificName formatted according to the conventions of the applicable nomenclaturalCode (http://rs.tdwg.org/dwc/terms/scientificNameAuthorship).
taxonRank	The taxonomic rank of the most specific name in the scientificName (http://rs.tdwg.org/dwc/terms/infraspecificEpithet).
taxonID	An identifier for the set of taxon information (http://rs.tdwg.org/dwc/terms/taxonID)
parentNameUsageID	An identifier for the name usage of the direct parent taxon (in a classification) of the most specific element of the scientificName (http://rs.tdwg.org/dwc/terms/parentNameUsageID).
resourceDescription	An account of the resource, including a data-paper DOI (http://purl.org/dc/terms/description)

## Additional information

The mollusks bivalvia taxonomy in Fauna Europaea proceeds from the CLECOM efforts (Falkner et al. 2001, Falkner et al. 2002). CLECOM (**C** heck **L** ist of **E** uropean **C** ontinental **M** ollusca) is a working group taking care about the taxonomy of continental (terrestrial and freshwater) mollusks. An equivalent working group on marine mollusks exists called CLEMAM. Both CLEMAM and CLECOM have been established at the 10th International Malacological Congress of the Unitas Malacologica in 1989, including prominent malacologists, taking care about the publishing of valid and invalid names of all European terrestrial and freshwater molluscs, according to the Code of Zoological Nomenclature.

## Figures and Tables

**Table 1.** Responsible specialists per family in Mollusca – Bivalvia.

**TAXONOMY**		**EUROPE**		
**FAMILY**	**SPECIALIST(S)**	**DATABASED SPECIES (Fauna Europaea)**	**TOTAL DESCRIBED SPECIES ​(information-gap)**	**TOTAL ESTIMATED SPECIES (knowledge-gap)**
Corbiculidae	Rafael Araujo	2	2	2
Dreissenidae	Rafael Araujo	5	7	7–10
Margaritiferidae	Rafael Araujo	2	2	2
Sphaeriidae	Rafael Araujo	30	34	35–40
Unionidae	Rafael Araujo	16	19	19–25
